# Physiological Action of Chloral Hydrate

**Published:** 1872-02

**Authors:** M. A. Byasson


### PHYSIOLOGICAL ACTION OF CHLORAL HYDRATE.

BY M. A. BYASSON.

In a note presented to the Académie des Sciences,* in anticipation of a more detailed memoir, the author gives some of the results of an investigation having reference specially to the physiological action of chloral hydrate. The conclusions, which differ from those of Dr. Oscar Liebreich, and have been founded upon the comparative action of chloroform, formate of soda, hydrate of chloral, trichloracetic acid and trichloracetate of soda upon frogs, rats and dogs—and incidentally of chloral hydrate upon men—are formulated as follows:

*Comptes Rendus, lxxii., 742.

1. The action of hydrate of chloral upon similar organisms is different from that of chloroform.

2. The action is peculiar to that body, and may be considered as the results of two products into which it is decomposed, principally upon contact with the blood—viz., chloroform and formic acid.

3. Trichloracetic acid and trichloracetate of soda differ from hydrate of chloral in their action upon the animal organism, since they both break up into chloroform and acetic acid.

A part of the chloroform formed by the action of the alkaline carbonates of the blood upon the hydrate of chloral is eliminated by the lungs; and a part of the formic acid is found in the urine in the shape of formate of soda. As a practical result of the experiments, the author found that he could distinguish three degrees, produced gradually and successively by increasing doses, but varying in individuals.

(1.) A feebly soporific action and slight sedative effect upon the sensitive nervous system, which may be accompanied by intervals of a peculiar agitation, similar to that produced by some dreams.

(2.) An energetic and powerful soporific action, with diminution of sensibility. Then follows a period of calm slumber of variable duration, but without apparent disturbance to the principal functions of life. By means of successive doses, administered when the effects of the previous ones have nearly disappeared, this slumber may be extended during a comparatively long time.

(3.) Anæsthetic action, with complete loss of sensibility and muscular power. Death has generally been found to follow when this stage has been reached, in consequence of the inability of the organism to sustain the increasing action of so large a quantity of the drug until its complete transformation and elimination.—*Phar. Journal and Transactions, December* 16, 1871.

